# The human urine virome in association with urinary tract infections

**DOI:** 10.3389/fmicb.2015.00014

**Published:** 2015-01-23

**Authors:** Tasha M. Santiago-Rodriguez, Melissa Ly, Natasha Bonilla, David T. Pride

**Affiliations:** ^1^Department of Pathology, University of California, San DiegoSan Diego, CA, USA; ^2^Department of Biology, San Diego State UniversitySan Diego, CA, USA; ^3^Department of Medicine, University of California, San DiegoSan Diego, CA, USA

**Keywords:** human microbiome, urinary tract infections, virome, virobiota, HPV, papillomavirus

## Abstract

While once believed to represent a sterile environment, the human urinary tract harbors a unique cellular microbiota. We sought to determine whether the human urinary tract also is home to viral communities whose membership might reflect urinary tract health status. We recruited and sampled urine from 20 subjects, 10 subjects with urinary tract infections (UTIs) and 10 without UTIs, and found viral communities in the urine of each subject group. Most of the identifiable viruses were bacteriophage, but eukaryotic viruses also were identified in all subjects. We found reads from human papillomaviruses (HPVs) in 95% of the subjects studied, but none were found to be high-risk genotypes that are associated with cervical and rectal cancers. We verified the presence of some HPV genotypes by quantitative PCR. Some of the HPV genotypes identified were homologous to relatively novel and uncharacterized viruses that previously have been detected on skin in association with cancerous lesions, while others may be associated with anal and genital warts. On a community level, there was no association between the membership or diversity of viral communities based on urinary tract health status. While more data are still needed, detection of HPVs as members of the human urinary virome using viral metagenomics represents a non-invasive technique that could augment current screening techniques to detect low-risk HPVs in the genitourinary tracts of humans.

## Introduction

The presence of microbes inhabiting the human urinary tract has generally been associated with urinary tract infections (UTIs), but recent studies have demonstrated that the urine has its own unique microbiota even in the absence of UTIs (Nelson et al., 2010; Siddiqui et al., 2011; Wolfe et al., 2012). Many of these microbiota may not be culturable using conventional culture techniques, but the presence of a diverse microbiota in human urine has highlighted the need to understand whether there is a role for these communities of microbes in urinary tract health or disease. Healthy female urinary microbiota often include organisms also identified in the vagina (Fouts et al., 2012; Wolfe et al., 2012; Hilt et al., 2014), while healthy male microbiota may resemble the vagina, gut, and skin (Nelson et al., 2010; Dong et al., 2011; Fouts et al., 2012). There are numerous lower urinary tract symptoms that often do not have known infectious etiologies, so understanding whether these conditions may be influenced by disturbances to the urinary microbiota is of substantial interest.

UTIs are among the most common urological disorders in the health care settings, and are diagnosed using a combination of both symptoms and culture tests. *Escherichia coli* is recognized as the most common etiological agent of UTI, responsible for more than 80% of reported cases in females (Foxman and Brown, 2003), but there are numerous other microbes capable of causing UTIs (Stamm, 2002; Pour et al., 2011; Volkow-Fernandez et al., 2012). While a threshold concentration of 10^3^ Colony Forming Units (CFU) per ml of urine accompanied with lower urinary tract symptoms is required for a UTI diagnosis, there are some pathogens that are not detected by standard microbiological culture techniques (Rubin et al., 1992). Disturbances to the urinary tract microbiota can be recognized in the presence of pathogens (Pearce et al., 2014), but it is not yet clear how long it takes for the urinary tract to recover its normal microbiota after both pathogen-mediated disturbances and antibiotic therapy.

While there is a substantial cellular microbiota indigenous to the human urinary tract, little is known about viruses. There are viral pathogens of the human urinary tract, such as adenovirus (Echavarria et al., 1998; Echavarria, 2008) and BK virus (Shinohara et al., 1993; Paduch, 2007; Egli et al., 2009), but these viruses generally are only pathogenic in subjects with compromised immune systems. There are other viruses such as Human papillomaviruses (HPVs) that inhabit human genital and rectal areas, and have previously also been found in urine (Pathak et al., 2014). Because HPVs can be associated with both warts and cancer in humans (Wylie et al., 2012), the diagnosis of its presence and particular subtypes associated with infection can be critical in the care of individual subjects who are infected. Previous studies have demonstrated that robust communities of cellular microbiota on human body surfaces are generally accompanied by communities of viruses. There are vast communities of viruses that inhabit the human oral cavity (Pride et al., 2012; Abeles et al., 2014; Ly et al., 2014), the human gut (Minot et al., 2011), the human respiratory tract (Willner et al., 2009), and human skin (Foulongne et al., 2012), which suggests that there likely would be communities of viruses that also are indigenous to the human urinary tract. One prior study identified a unique viral community in the subgingival crevice of individuals with severe periodontal disease (Ly et al., 2014), which suggests that viruses could play a role in health and disease. There are many subjects with lower urinary tract symptoms, with no known cellular microbial etiologies, yet viral communities in these subjects are completely unexplored (Enerly et al., 2013).

We sought to decipher whether there is a population of viruses indigenous to the human urinary tract and whether urine viral community membership may be affected by urinary tract health status. Our goals were to: (1) identify and quantify viral populations in human urine, (2) determine whether trends in urine bacterial biota might also be reflected in the virobiota of urine, (3) elucidate whether the presence of urinary pathogens affects viral community membership, (4) decipher whether human viruses populate the urinary tract, or whether viruses encountered are primarily bacteriophage, and (5) characterize differences between the virobiota of the male and female urinary tracts.

## Materials and methods

### Human subjects and culture conditions

Human subject involvement in this study was approved by the University of California, San Diego Administrative Panel on Human Subjects in Medical Research. The study was certified as category 4 exempt, which includes research involving the collection or study of existing data, documents, records, pathological specimens, or diagnostic specimens, if the information is recorded in such a manner that subjects cannot be identified, directly or through identifiers linked to the subjects. We sampled the urine from 20 human subjects, with 10 having urinary tract infections, and another 10 testing negative for UTIs. None of the female subjects had prior abnormal PAP smears and therefore had never been tested for the presence of cervical HPV. None of the male subjects had anal PAP smears and were not previously tested for HPV. No subjects reported any prior history of anal or genital warts. The diagnosis of UTI was based on the clinical laboratory standards institute definition, and includes the presence of ≥10^3^ CFU of bacteria and ≥10 leukocytes per high powered field (Stamm, 1983). All specimens were planted on Sheep's blood agar, McConkey, and Chocolate agar plates. Each of the patients in this study was symptomatic and had at least 10^3^ CFU/ml of bacteria in their urine specimens. Bacteria were identified to the species level using Matrix-Assisted Laser Desorption Ionization Time of Flight mass spectrometry, which is a mass spectrometry technique for the identification of bacteria with sensitivity and specificity similar to that of conventional biochemical techniques (Neville et al., 2011). For the *E. coli* isolates, their identification also was verified by the presence of lactose fermentation and mobility to ensure that they could not be *Shigella* species. Urine specimens were processed within 2 h of their collection and the bacteria were identified within 24 h of their collection. All specimens from both UTI+ and UTI− subjects had their viromes processed within 48 h of their collection. All cultures from UTI− subjects were held for a minimum of 72 h to ensure they were truly negative. All urine specimens were stored at 4°C prior to processing of viromes.

### Virome preparation and sequencing

Urine samples were processed according to our previously described protocols for processing viruses from saliva (Pride et al., 2012). Urine (1 ml) was filtered sequentially using 0.45 and 0.2 μm filters (VWR, Radnor, PA) and purified on a cesium chloride density gradient, with the fraction corresponding to most known bacteriophage (Murphy et al., 1995) retained. The purified virions were further purified on Amicon YM-100 protein columns (Millipore, Inc., Bellerica, MA), treated with DNase I, and their DNA purified using a Qiagen UltraSens virus kit (Qiagen, Valencia, CA). DNA then was amplified using GenomiPhi V2 MDA amplification (GE Healthcare, Pittsburgh, PA), fragmented to 200–400 bp using a Bioruptor (Diagenode, Denville, NJ), and libraries created using the Ion Plus Fragment Library Kit. Libraries then were sequenced using 314 chips on an Ion Torrent Personal Genome Machine (PGM; Life Technologies, Grand Island, NY) (Rothberg et al., 2011) producing an average read length of approximately 206 bp.

### Virome analysis

We trimmed the resulting reads according to modified Phred scores of 0.5 using CLC Genomics Workbench 4.65 (CLC bio USA, Cambridge, MA), removed any low complexity reads (where >25% of the length were due to homopolymer tracts), and removed any reads with substantial length variation (<50 nucleotides or >300 nucleotides) or ambiguous characters prior to further analysis. Each virome was screened for contaminating bacterial and human nucleic acids using BLASTN analysis (E-score <10^−5^) against the Ribosomal Database Project 16S rRNA database (Cole et al., 2009), and the human reference database available at ftp://ftp.ncbi.nlm.nih.gov/genomes/H_sapiens/. Any reads homologous to human sequences were removed prior to further analysis. GC content variation among contigs was assessed using Box and Whiskers plots created using Microsoft Excel 2007 (Microsoft Corp., Redman, WA). Remaining reads were assembled using CLC Genomics Workbench 4.65 based on 98% identity with a minimum of 50% read overlap, which were more stringent than criteria developed to discriminate between highly related viruses (Breitbart et al., 2002). Because the shortest reads were 50 nucleotides, the minimum tolerable overlap was 25 nucleotides, and the average overlap was no less than 103 nucleotides depending on the characteristics of each virome. The consensus sequence for each contig was constructed according to majority rule, and any contigs <200 nucleotides or with ambiguous characters were removed prior to further analysis. Contigs were annotated using BLASTX against the NCBI Non-redundant (NR) database with an E-score cutoff value of 10^−5^. Specific viral homologs were determined by parsing BLASTX results for known viral genes including replication, structural, transposition, restriction/modification, hypothetical, and other genes previously found in viruses for which the E-score was at least 10^−5^. Each individual virome contig was annotated using this technique; however, if the best hit for any portion of the contig was to a gene with no known function, lower level hits were used as long as they had known function and still met the E-score cutoff. The annotation data were compiled for each subject and used to determine the relative proportions of assembled contigs that contained viral homologs. Analysis of shared homologs present in each virome was performed by creating custom BLAST databases for each virome, comparing each database with all other viromes using BLASTN analysis (E-score <10^−10^). Beta diversity between viromes was determined using binary Sorensen distances, and was determined by randomly sampling 1000 contigs between viromes. Principal coordinates analysis (PCOA) was performed with the estimated distances using Qiime (Caporaso et al., 2010b). Read mappings of viromes to a database of phage (www.phantome.org; ftp://ftp.ncbi.nih.gov/genomes/Viruses/) and a database of viruses (ftp://ftp.ncbi.nih.gov/genomes/Viruses/) was performed using CLC Genomics Workbench 4.65 (CLC bio USA, Cambridge, MA), and were mapped using 98% identity over a minimum of 50% of the read length. Virome sequences are available for download in the MG-RAST database (metagenomics.anl.gov/) under the project “UrineViromeProject,” or under project #9680.

### Viral diversity

To measure alpha diversity in the viral communities, we utilized a technique termed the Homologous Virus Diversity Index (HVDI). The technique is based on finding high levels of homology amongst contigs within viromes that likely belong to the same virus but were placed into separate contigs due to the limitations of the assembly process (Abeles et al., 2014). Virome reads were assembled using 98% identify over a minimum of 50% of the read length using CLC Genomics Workbench 4.65 (CLC bio USA, Cambridge, MA), and the resulting contig spectra utilized as the primary input for the index. We created custom nucleotide BLAST databases for each subject that contained all their contigs. We then used BLASTN analysis to find high levels of homology (E-score <10^−20^) between different contigs within the same subject. We accepted only high levels of homology that spanned at least 50% of the length of the shorter contig being compared. All contigs in each subject were treated as nodes and those contigs that had high homology to other contigs in the same subject were added to a network by directing edges between the nodes. After evaluating homologies among all intra-subject contigs, networks formed from directed edges/nodes were assigned to individual viruses and nodes with no associations were considered singular viruses. For each resulting network, we added the number of reads assigned to each node on the network and the combined number of reads was used to represent the relative abundance of the virus represented by that network. The relative abundances of all viruses were calculated using this technique, and a new contig spectra representing the viral population in each subject was formed. The contig spectra from each subject then were used as surrogates for population structures and input directly into the Shannon Index (Gotelli and Colwell, 2001) to estimate diversity. We also utilized the relative numbers of viral contigs created from only a single read (singleton) or from only two reads (doubleton) as input for the chao1 index (Chao, 1984). The results of the corrected contig spectra in each case were compared with uncorrected contig spectra, and in each case resulted in an approximate 10% reduction in diversity calculations.

### Quantitative PCR

We designed primers HPV49F (GTGATTCAAATGCAAGGG) and HPV49R (TTTACAATCTCAGACCAGTG) to target HPV genotype 49 and HPV178F (TGTTTGGAAGGGATCTAGT) and HPV178R (TCATTAAAAAGCGCCAGG) to target HPV genotype 178. Primers for both genotypes were based on early gene 1 sequences. We performed quantitative PCR in 20 μl reactions using 10 μl of SYBR Select Master Mix (Life Technologies, Grand Island, NY), 1 μl of template viral DNA (10 ng/μl), 7 μl of ultrapure water, and 1 μl (0.5 pmol) of each of the forward and reverse primers. The following cycling parameters were used: an initial denaturation at 95°C for 2 min, followed by 35 cycles at 95°C for 15 s, annealing at 54°C for 15 s, and final extension at 60°C for 1 min on an Eppendorf Mastercycler Realplex2. Each reaction was run in triplicate and results were only considered positive if each replicate had a *Ct*-value =35 with a product at the expected size. One of the products was verified by Sanger sequencing of the PCR amplicon and produced a product identical to the reads matching HPV already found.

### Analysis of 16S rRNA

Genomic DNA was prepared from the urine of each subject and time point using the Qiagen QIAamp DNA MINI kit (Qiagen, Valencia, CA). Each sample was subjected to a bead beating step prior to nucleic acid extraction using Lysing Matrix-B (MP Bio, Santa Ana, CA). We amplified the bacterial 16S rRNA V3 hypervariable region using the forward primer 341F (CCTACGGGAGGCAGCAG) fused with the Ion Torrent Adaptor A sequence and one of 23 unique 10 base pair barcodes, and reverse primer 514R (ATTACCGCGGCTGCTGG) fused with the Ion Torrent Adaptor P1 from the urine of each subject (Whiteley et al., 2012). PCR reactions were performed using Platinum PCR SuperMix (Invitrogen, Carlsbad, CA) with the following cycling parameters: 94°C for 10 min, followed by 30 cycles of 94°C for 30 s, 53°C for 30 s, 72°C for 30 s, and a final elongation step of 72°C for 10 min. Resulting amplicons were purified on a 2% agarose gel stained with SYBR Safe (Invitrogen, Carlsbad, CA) using the MinElute PCR Purification kit (Qiagen, Valencia, CA). Amplicons were further purified with Ampure beads (Beckman-Coulter, Brea, CA), and molar equivalents were determined for each sample using a Bioanalyzer 2100 HS DNA Kit (Agilent Technologies, Santa Clara, CA). Samples were pooled into equimolar proportions and sequenced on 314 chips using an Ion Torrent PGM according to manufacturer's instructions (Life Technologies, Grand Island, NY) (Rothberg et al., 2011). Resulting sequence reads were removed from the analysis if they were <130 nucleotide, had any barcode or primer errors, contained any ambiguous characters, or contained any stretch of >6 homopolymers. Sequences were assigned to their respective samples based on their 10 nucleotide barcode sequence, and were analyzed further using the Qiime pipeline (Caporaso et al., 2010b). Briefly, representative OTUs from each set were chosen at a minimum sequence identity of 97% using UClust (Edgar, 2010) and aligned using PyNast (Caporaso et al., 2010a) against the Greengenes database (Desantis et al., 2006). Multiple alignments then were used to create phylogenies using FastTree (Li and Godzik, 2006), and taxonomy was assigned to each OTU using the RDP classifier (Wang et al., 2007; Price et al., 2009). PCOA was performed based on Beta Diversity using weighted Unifrac distances (Lozupone et al., 2006). Alpha diversity based on the Shannon Index and the chao1 index also were performed using the Qiime pipeline. Differences in the relative abundances of taxa between subject groups were determined using student's *t*-test. 16S rRNA sequences are available for download in the MG-RAST database (metagenomics.anl.gov/) under the project “UrineViromeProject,” or under project #9680.

### Statistical analysis

To assess whether viromes had significant overlap between subjects and subject groups, we performed a permutation test based on resampling (10,000 iteration) (Robles-Sikisaka et al., 2013, 2014; Abeles et al., 2014; Ly et al., 2014; Naidu et al., 2014). We simulated the distribution of the fraction of shared virome homologs from two different time points within individual subjects that were randomly chosen across all time points. For each set, we computed the summed fraction of shared homologs using 1000 random contigs between randomly chosen individual time points within different subjects, and from these computed an empirical null distribution of our statistic of interest (the fraction of shared homologs). The simulated statistics within each subject group were referred to the null distribution of inter-group comparisons, and the *p*-value was computed as the fraction of times the simulated statistic for the each exceeded the observed statistic. An identical analysis was performed at the OTU level for the16S rRNA taxonomic assignments. For analysis of sex specific characteristics within the viromes, a randomly chosen subject and time point from the male sex was compared with a randomly chosen subject and time from the female sex to determine the null distribution of fraction of shared contigs based on opposite sexes. We then estimated the fraction of shared homologs from randomly chosen subjects and time points within each sex and compared with the empirical null distribution from simulated inter-sex values. We estimated the *p*-value based on the fraction of times the intra-sex statistic exceeded that for the observed statistic.

## Results

### Human subject characteristics

We recruited 20 human subjects, 10 that were diagnosed with UTIs and 10 with negative urine cultures (Table 1). The diagnosis of UTI was made in the urine based upon the presence of ≥10 leukocytes per high powered field along with ≥10^3^ CFU of bacteria. Of those 10 subjects with UTI's, eight grew *E. coli*, one grew *Enterococcus faecium*, and one grew *Acinetobacter baumanii* complex from their urine. Five of the subjects were male and five were female in both arms of the study. We processed viromes from the urine in each subject within 24 h of their collection to ensure that viral structure and nucleic acid integrity would not be affected by processing times.

**Table 1:** **Study subjects**.

**Subjects**	**Sex**	**Age**	**Symptoms**	**Immuno compromised^a^**	**Organism**	**Catheter^b^**
**NEGATIVE URINE CULTURES**						
URN1	Male	94	None	No	None	No
URN2	Male	57	Sepsis^c^	Yes	None	Yes
URN6	Male	51	Pain in prostate area	No	None	Yes
URN9	Male	47	Pain with urination	No	None	No
URN10	Female	27	Vaginal bleeding	No	None	No
URN11	Female	31	Pain with urination	No	None	No
URN12	Female	25	Abdominal pain	No	None	No
URN13	Female	51	Urine odor	Yes	None	No
URN15	Female	54	Sepsis^c^	No	None	Yes
URN16	Male	72	None	No	None	No
**POSITIVE URINE CULTURES**						
URP1	Male	62	Urinary retention	No	*E. coli*	No
URP2	Female	34	Sepsis^c^	Yes	*E. coli*	Yes
URP3	Female	50	Stroke	No	*E. coli*	No
URP4	Male	65	None	No	*E. coli*	Yes
URP6	Female	18	Dysuria	No	*E. coli*	No
URP7	Female	22	Autonomic instability	No	*E. faecium*	Yes
URP9	Male	52	Sepsis^c^	Yes	*E. coli*	No
URP10	Male	58	Sepsis^c^	Yes	*Acinetobacter*	Yes
URP12	Male	78	Sepsis^c^	Yes	*E. coli*	Yes
URP14	Female	69	Dysuria	Yes	*E. coli*	No

a *Includes subjects with cancer and organ transplants, and those taking immunosuppressive medications such as steroids*.

b *Includes subjects with catheters in their bladders and a single subject with a nephrostomy*.

c *Potentially life threatening complication of infection resulting in severe inflammation and blood pressure drops*.

### Epifluorescence microscopy

Because viral communities in human urine have not previously been characterized, we first utilized epifluorescence microscopy to determine whether viral communities were present and to estimate their concentrations. We found that there were approximately 10^7^ virus-like particles (VLPs) per ml of urine present in each of the subjects in this study (Supplemental Figure 1). No differences in VLP concentrations were observed between subjects with or without UTIs. Comparatively, there generally are 10^8^ VLPs per ml of saliva (Pride et al., 2012) and 5 × 10^8^ VLPs per ml of human feces (Haynes and Rohwer, 2011). The relatively low concentrations of viruses in urine may reflect the lower relative abundance of the microbiota in the human urinary tract when compared to the oral cavity and the gut. These data suggest that there are communities of viruses present in the human urinary tract whose relative abundance are not affected by the presence of urinary pathogens.

### Urine virome sequence characteristics

We isolated viral communities from the urine of our cohort using our previously described methods (Robles-Sikisaka et al., 2013, 2014; Abeles et al., 2014; Ly et al., 2014; Naidu et al., 2014). We sequenced a total of 14,542,349 reads, 6,734,864 from subjects with UTIs and 7,807,485 from subjects without UTIs (Supplemental Table 1). The mean number of reads per subject was 727,117, with a mean length of 206 nucleotides, and a mean GC content of 40.1%. Viromes were screened for contaminating cellular DNA by BLASTN (E-score <10^−5^) analyses against the Ribosomal Database Project 16S rRNA database (Cole et al., 2009) and a Human Reference Genome database (ftp://ftp.ncbi.nlm.nih.gov/genomes/H_sapiens/). None of the viromes had identifiable 16S rRNA, and 0.42% of the reads were homologous to human DNA. Reads homologous to human DNA were removed prior to further analysis. We assembled the viral reads into contigs, as the larger contigs generally result in more productive BLAST searches. We obtained an average of 2285 contigs per subject with a mean length of 1209 nucleotides and a mean GC content of 41.4%. No significant differences were observed in GC content between subjects with and without UTIs (Supplemental Figure 2).

### Detection of human papillomaviruses (HPVs)

**Figure 1:**
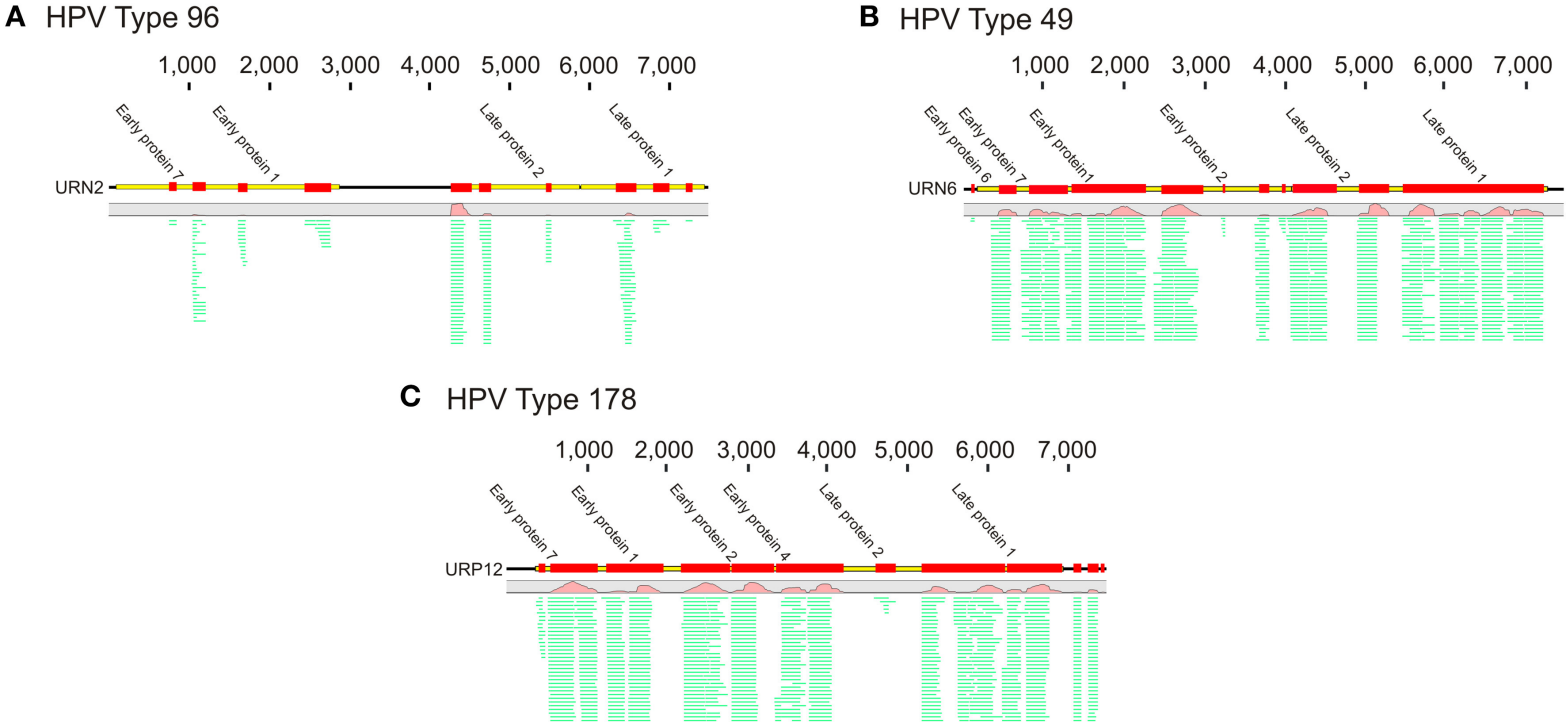
**Read mappings of select viromes to Human papillomaviruses (HPVs). (A)** Represents URN2 mapping to HPV Type 96, **(B)** represents URN6 mapping to HPV Type 49, and **(C)** represents URP12 mapping to HPV Type 178. The relative coverage of each contig is represented, along with annotated open reading frames (ORFs) above each box. The portions of the contigs identified are represented by the colored boxes for each subject. The length of each contig is denoted at the top of each panel. The relative proportions of reads mapping to each virus are represented in green.

**Figure 2:**
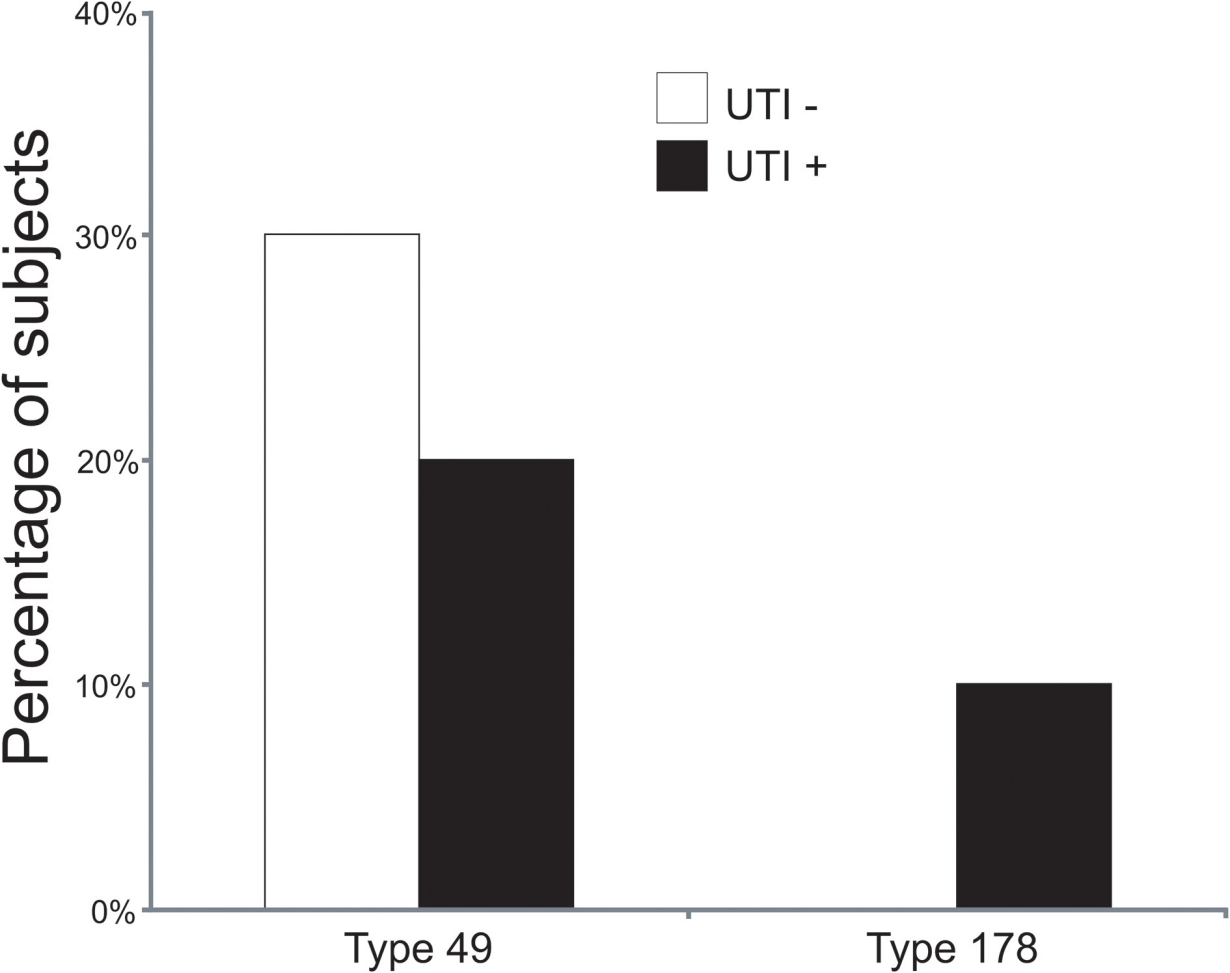
**Bar graphs of the percentage of subjects with detectable HPV Types 49 and 178 by quantitative PCR**. White bars indicate those subjects with negative urine cultures, and black bars represent subjects with urinary tract infections.

To decipher whether there may be individual viruses in this cohort that have high similarity to known viruses, we mapped the reads from each virome to the NCBI virus database. We found reads in both UTI+ and UTI− subjects that matched herpesviruses, polyomaviruses, and human papillomaviruses. The HPVs were much more prevalent than any other eukaryotic viruses identified in these subjects, and many of the viromes had reads that mapped specifically across most of the genomes of the HPVs. Nineteen of the 20 (95%) subjects mapped across different HPV viromes, which included all UTI− subjects and nine of 10 (90%) of the UTI+ subjects (Supplemental Table 2). For subject URN2, 1.7% (14,647 reads) of the reads mapped to HPV Type 96 (Figure 1A), and in subject URN6, 1.8% (13,948 reads) of the virome reads mapped to HPV Type 49 (Figure 1B). Different reads from these subjects also mapped to HPV Types 92, and 121 (data not shown). In subject URP12, 3.7% (25,389 reads) of the reads mapped to HPV Type 178 (Figure 1C) and Type 121 (data not shown). None of the subjects participating in this study had previously been diagnosed with genital warts or HPV infections, so the presence of HPV in the urine of these subjects suggests that urine may be a reasonable specimen type for screening for urogenital HPV infections. None of the females had previously had abnormal PAP smears or cervical HPV testing, but the types of HPVs identified by commercially available tests would not have identified the genotypes present in this study. To confirm the presence of some HPV types in the urine, we developed primers for HPV Types 49 and 178, as several subjects had reads that mapped specifically to these viruses. We confirmed the presence of nucleic acids matching HPV Type 49 in the urine of five of the 20 subjects (Supplemental Figure 3), while 10% of the subjects had nucleic acids matching HPV Type 178 (Figure 2).

### Identification of urinary bacteriophage

Viromes were also mapped against a phage database. We found many viral reads that mapped to individual viruses, however, many only mapped to single genes often involved in viral replication. We did find some that mapped across much of the genomes of known viruses, including subject URN2 mapping to Lambda phage (Supplemental Figure 4A), URN12 mapping to *Staphylococcus* phage PH15 (Supplemental Figure 4B), URP1 mapping to *E. coli* phage phiV10 (Supplemental Figure 4C), and URP2 mapping to *Enterococcus* phage phiFL4A (Supplemental Figure 4D).

**Figure 3:**
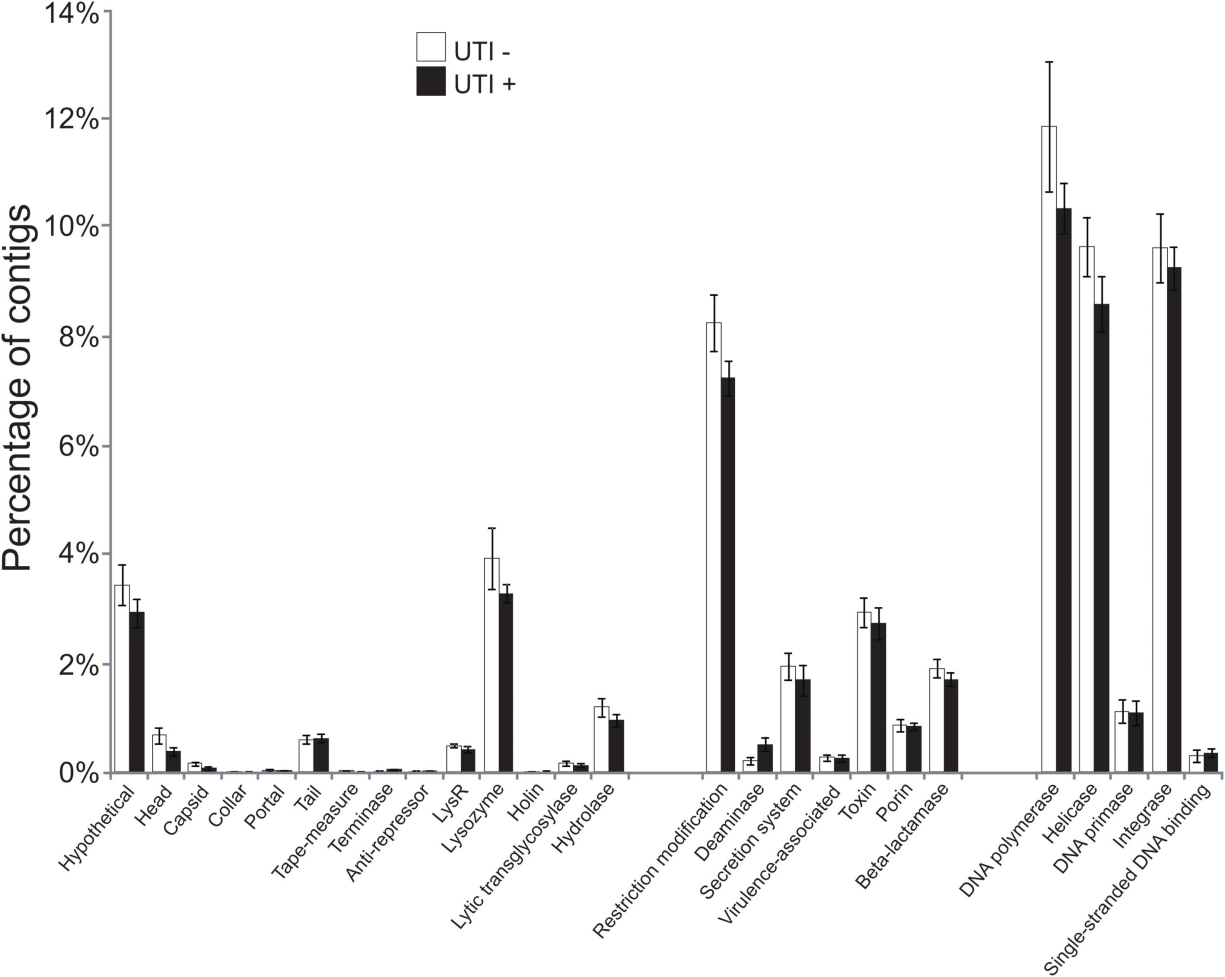
**Bar graphs of the mean percentages of contigs (± standard error) with viral homologs in the NR database from all of the subjects**. White bars indicate those subjects with negative urine cultures, and black bars represent subjects with urinary tract infections.

To further aid in deciphering the contents of the viromes, we subjected all the assembled contigs to BLASTX analysis against the NCBI NR database to identify homologous sequences. We identified numerous contigs with homologous sequences in the NR database, with most of those sequences homologous to phage. Approximately 27 percent of the contigs were homologous to known viruses, with the majority (>99%) representing bacteriophage (Supplemental Figure 5). We identified contigs with homologs to viral hypothetical proteins, structural genes (head, capsid, collar, portal tail, tail fibers), restriction modification enzymes, virulence and DNA replication and integration genes (DNA polymerases, helicases, integrases, and primases) (Figure 3). No significant differences were noted for the viral gene categories in association with urological health status. The high proportions of integrases identified (Figure 3) suggests that viruses with primarily lysogenic lifestyles were abundant in the urine viromes.

### Alpha diversity amongst urinary microbiota

**Figure 4:**
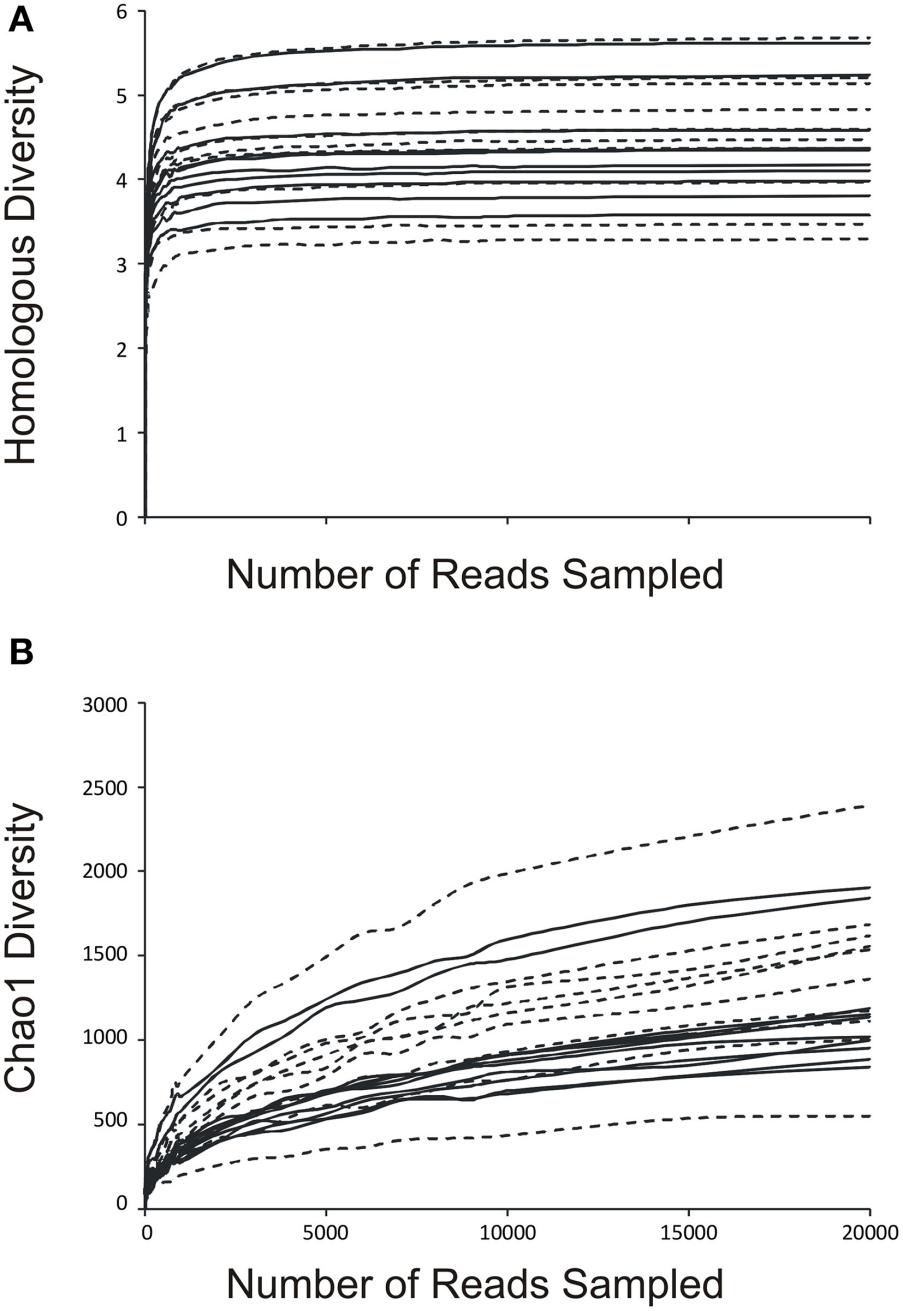
**Rarefaction analysis based on the Homologous Virus Diversity Index using the Shannon Index (A) and the chao1 Index (B) for urine viral communities**. The index values are shown on the y-axis and the number or reads sampled are shown on the x-axis. White boxes indicate those subjects with negative urine cultures, and black triangles represent subjects with urinary tract infections. Dashed lines indicate those subjects with negative urine cultures, and solid lines represent subjects with urinary tract infections.

We determined the alpha diversity present in the urinary viromes to determine if significant differences exist between subjects based on urinary tract health status and as a measure of the relative adequacy of our sequencing depth. We performed rarefaction analysis on the urinary viromes based on the Homologous Virus Diversity Index (HVDI) (Santiago-Rodriguez et al., under review) to determine the adequacy of our sequencing depth. We first calculated the HVDI based on the Shannon Index (Figure 4A), which indicated that most of the diverse viruses present in the community had been sampled with relatively minimal sequencing efforts. The index calculated based on the chao1 index (Figure 4B) approached asymptote for most urine samples, which corroborates that the sequencing depth for these urinary viromes was adequate.

**Figure 5:**
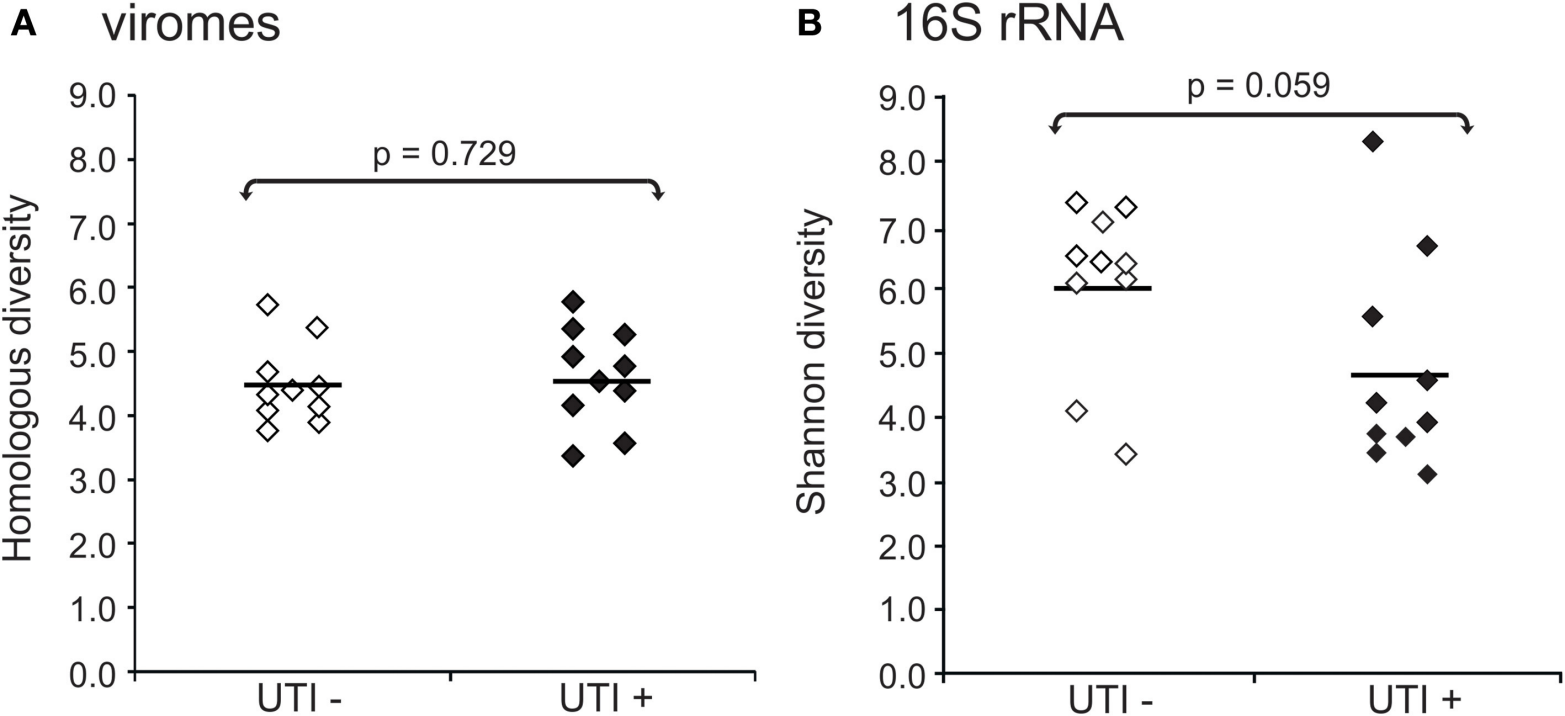
**Plots of alpha diversity for urine viromes (A) and bacteria biota using 16S rRNA (B)**. Viral diversity was determined using the Homologous Virus Diversity Index based on the Shannon Index, and bacterial diversity determined using the Shannon Index. White diamonds indicate those subjects with negative urine cultures, and black diamonds represent subjects with urinary tract infections.

We also characterized the bacterial communities through analysis of the 16S rRNA V3-hypervariable region. We compared the alpha diversity present in bacterial and viral communities using the Shannon index for the bacterial biota. There were no observed differences in viral community diversity based on urinary tract health status (Figure 5A) (*p* = 0.729); however, bacterial diversity was substantially lower in subjects with UTIs (Figure 5B) (*p* = 0.059), probably reflecting the relative abundance of the urinary pathogens present. That viral diversity is unaffected despite differences in bacterial biota, suggests that the urinary pathogens may not contribute substantially to overall viral diversity.

### Beta diversity in urinary microbiomes

**Figure 6:**
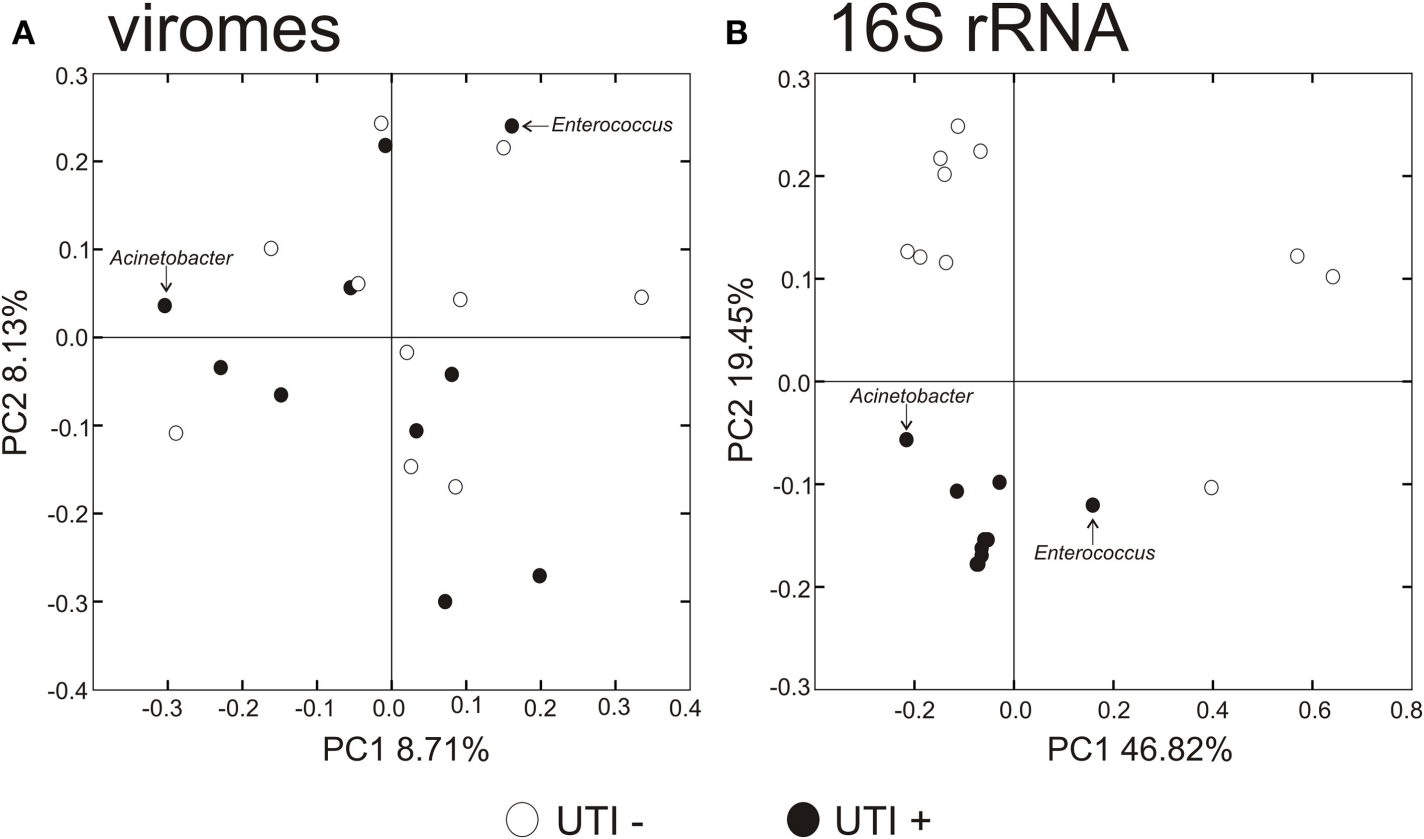
**Principal coordinates analysis of beta diversity of the urine viromes (A), and bacterial biota using 16S rRNA (B)**. White circles indicate those subjects with negative urine cultures, and black circles represent subjects with urinary tract infections.

To decipher whether there may exist differences in virome community membership based on urinary tract health status, we compared beta diversity amongst the viromes and visualized patterns of variation present using principal coordinates analysis (Figure 6A). We did not observe any patterns of variation in the viromes that were attributable to health status. We did, however, identify substantial differences when examining the bacterial biota, where those subjects with UTIs formed a somewhat homogenous cluster (Figure 6B). We previously have identified host-sex specific characteristics of viral communities in human saliva (Abeles et al., 2014), and we tested whether there may be specific traits of the virobiota of urine that might also be host-sex specific. For the viral communities, no host-sex specific differences were identified for viral communities (Supplemental Figure 6).

We utilized a permutation test to determine whether the proportion of homologous viral contigs among subjects with UTIs was greater than would be expected to occur by chance (Abeles et al., 2014; Ly et al., 2014). Similar to the results for the PCOA (Figure 6A), there were no significant differences observed for virome community membership by urinary tract health status (Table 2). While the differences were not statistically significant, there were more shared bacterial OTUs amongst the subjects based on their urinary tract health status. There were no significant differences for viromes or the bacterial biota based on host sex.

**Table 2:** **Viral homologs and shared 16S OTUs within and between subject groups**.

	**Percent homologous within group^a^**	**Percent homologous between groups^a^**	***p*-Value^b^**
**BY URINE HEALTH STATUS**			
**Viromes**			
UTI−	24.14 ± 4.86	23.73 ± 4.34	0.453
UTI+	23.79 ± 4.51	23.70 ± 4.32	0.512
**16S rRNA**			
UTI−	84.02 ± 19.05	73.36 ± 27.23	0.343
UTI+	87.29 ± 16.36	73.60 ± 27.12	0.301
**BY SEX**			
**Viromes**			
Male	23.28 ± 3.93	23.64 ± 4.44	0.524
Female	24.66 ± 4.63	23.74 ± 4.48	0.424
**16S rRNA**			
Male	88.47 ± 10.82	75.42 ± 25.99	0.377
Female	76.55 ± 27.72	76.26 ± 25.48	0.470

a *Based on the mean of 10,000 iterations. Thousand random contigs were sampled per iteration*.

b *Empirical p-value based on the fraction of times the estimated percent homologous contigs or shared OTUs for each group exceeded that between groups*.

### Blast hits and bacterial taxonomies

**Figure 7:**
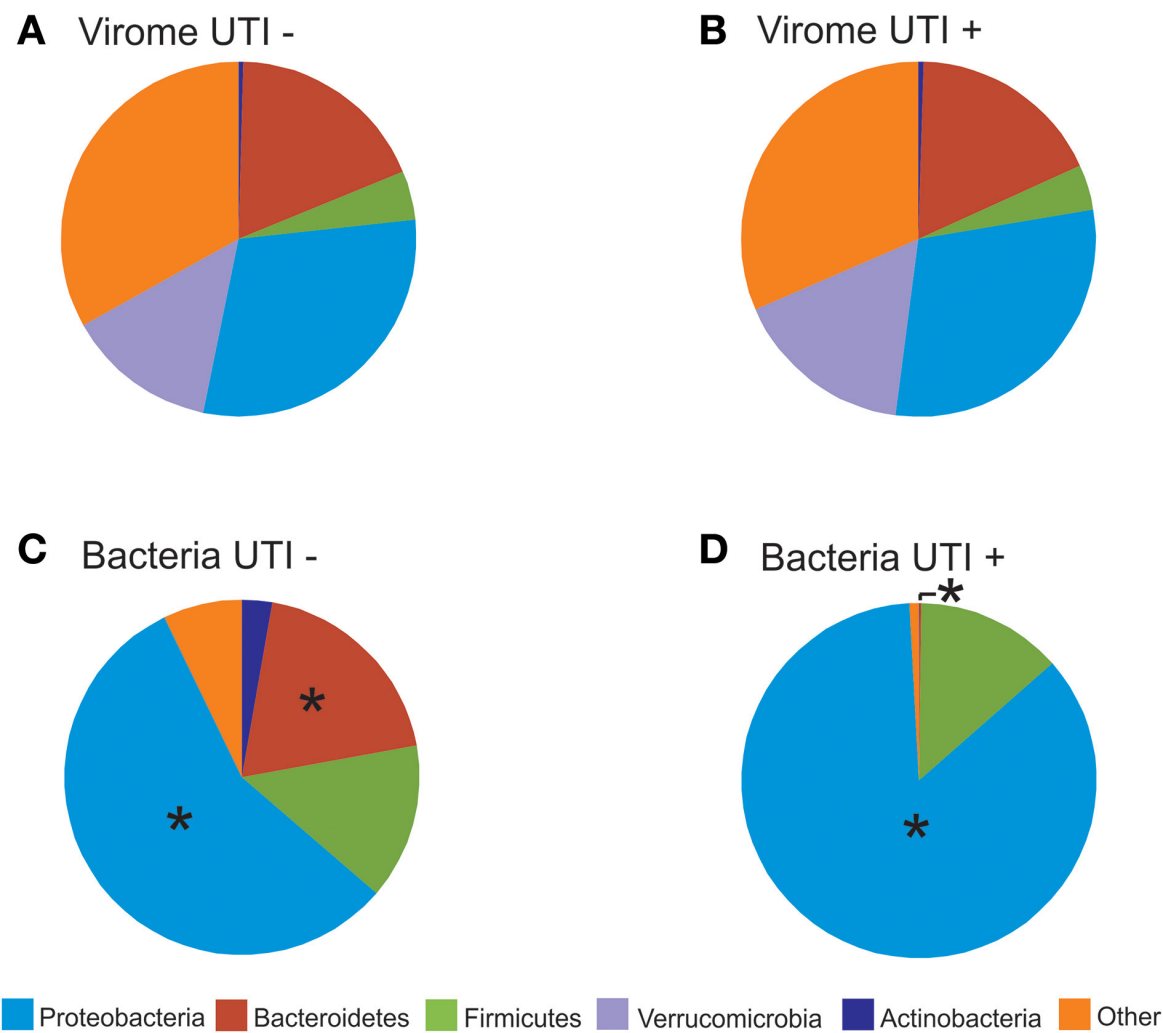
**Pie charts of phage BLASTX hits (A,B) and bacterial taxonomy using 16S rRNA (C,D)**. **(A)** and **(C)** represent subjects with negative urine cultures, and **(B)** and **(D)** represent subjects with urinary tract infections. The “^*^” represents significant differences (*p* = 0.05) between the proportions of different phyla represented between UTI+ and UTI− subjects.

We characterized the BLASTX hits for each virus homolog by the taxonomy of the host bacteria at the Phylum level. While this type of characterization does not provide accurate identification of the putative hosts for each viral homolog, it can be utilized comparatively to understand whether virome homologs might reflect 16S rRNA taxonomies. By characterizing the viral communities in this manner, we find that BLASTX hits to Proteobacteria are most predominant, followed by Bacteroidetes, Actinobacteria, Verrucomicrobia, and Firmicutes (Figures 7A,B and Supplemental Figure 7). The BLASTX hits were similar regardless of urinary tract health status, which again suggests that urinary tract health does not determine virome community membership. Trends in urine viral communities were not represented in the bacterial taxonomies (Figures 7C,D).

## Discussion

Human body surfaces are inhabited by indigenous microbial communities whose role in health and disease are just beginning to be elucidated. The urine was once believed to constitute a sterile environment, but studies now have determined that both the male and female genitourinary tracts are home to viable bacterial communities. We hypothesized that because these communities are home to cellular microbes that they would also have indigenous communities of viruses, and this study is the first to demonstrate that urine indeed has a robust community of viruses. We found that there were approximately 10^7^ VLPs in the urine of UTI+ and UTI− subjects, which is an order of magnitude lower than we have previously found in human saliva (Pride et al., 2012). This may reflect differences in the relative abundances of bacteria in the urine compared to the oral cavity, as the healthy human oral cavity has many cultivable bacterial biota, while few are found in healthy urine. While many of the viruses identified were bacteriophage, as has been demonstrated in many prior studies (Breitbart et al., 2003; Willner et al., 2009, 2011; Reyes et al., 2010; Minot et al., 2012; Pride et al., 2012; Ly et al., 2014; Naidu et al., 2014; Robles-Sikisaka et al., 2014), the presence of HPVs, which are eukaryotic viruses of the human host, suggests that human urine is a complex community with numerous different indigenous virus genotypes. Our methods to characterize viral communities generally have not identified many enveloped viruses; however, we previously have identified herpesviruses in the human oral cavity using these methods (Pride et al., 2012; Ly et al., 2014), so the extent of any biases imposed by using CsCl density gradient centrifugation remains unclear. We focused our analysis of phage on the contigs assembled from the virome reads because the longer contigs allowed for more productive searches for homologous sequences, but 73% of the contigs had no identifiable homologs. The analysis of contigs did not identify many eukaryotic viruses because their relatively low abundance and probable low diversity resulted in few assembled contigs.

Many of our studies have found few eukaryotic viruses in the oral microbiome when compared to the abundance and diversity of bacteriophage (Pride et al., 2012; Robles-Sikisaka et al., 2013; Abeles et al., 2014; Ly et al., 2014), suggesting that eukaryotic viruses represent a relatively small proportion of the human virome (Wylie et al., 2012). In the present study we showed that HPVs comprise a portion of the urine virome in some subjects regardless of urinary tract health status. We were able to detect HPV Types in 19 of the 20 (95%) subjects studied, suggesting that HPV may be relatively common urinary virobiota. The HPV genotypes identified in this study have not been associated with high risks for cervical carcinomas, but Type 9 identified in some subjects may be associated with the development of cutaneous cancers (Andersson et al., 2012). Other subjects mapped to Type 49, which has been isolated from warts in renal transplant patients (Favre et al., 1989), and Type 178, which was isolated from skin adjacent to actinic keratosis (Johansson and Forslund, 2014; Martin et al., 2014). Our results are among the first to demonstrate the presence of these HPV genotypes in urine, presenting the opportunity to further investigate their role in urological health and diverse urological diseases. Unfortunately, the cohort of subjects studied was not ideal for characterizing potential pathogenic effects of the HPV genotypes identified because none had prior cystoscopies, culposcopies, or body imaging to document bladder or cervical pathology. Because the HPV genotypes in this study were not high risk genotypes, cystoscopies and culposcopies were not clinically indicated. Typical protocols for identifying HPV in women are based on the presence of atypical cervical pathology (Nobbenhuis et al., 1999), although primary HPV screening is now becoming more prevalent (Whitlock et al., 2011). None of the females in this study had prior abnormal PAP smears, and thus had not been tested for HPV infections. HPV diagnostic tests in clinical laboratories test for high risk genotypes (Ronco et al., 2010), and would not have identified the genotypes found in this study. We believe that most of the genotypes we identified probably may be associated with genital warts, although none of the subjects previously reported having genital warts.

We characterized the human urinary virome in association with UTIs, as they are the most frequent cause of genitourinary morbidity in adults (Resnick and Older, 1997; Foxman, 2003). It was somewhat surprising that the bacterial communities were significantly altered in UTI+ subjects (Figures 6, 7), while there were no obvious differences observed in urine viral communities. In the human oral cavity, we previously found that both bacterial and viral communities were altered in subjects with moderate/severe periodontal disease (Ly et al., 2014). However, even when viral communities are composed mostly of bacteriophage, their membership does not necessarily reflect that of their host communities, likely due to different dynamic relationships present for different host/phage pairs (Pride et al., 2012). We believe that the lack of differences observed in UTI+ subjects may be secondary to the pathogens in these infections contributing relatively few viruses to the urinary microbiota. Further work would be necessary to decipher the contribution of phage from the pathogens in these subjects' urine microbiomes.

Similar to previous studies, there were identifiable differences in bacteria taxa in association with urological health status (Pearce et al., 2014). Urine from UTI− subjects exhibited higher bacterial diversity when compared to UTI+ subjects (Figure 5B). These differences were not observed for viral communities (Figure 5A), as the pattern of BLASTX homologs in UTI+ and UTI− subjects suggests that much of the viral community is conserved regardless of urinary tract health status (Figures 7A,B). Recent work has demonstrated that phage are capable of attaching to mucosal surfaces (Barr et al., 2013), so it is possible that the viruses observed in UTI+ patients could reflect viruses swept into the urine from mucosal surfaces rather than actively replicating urine viruses. Study of the urinary tract transcriptome in a cohort of UTI+ and UTI− patients could help elucidate whether the viruses observed were actively transcribing/replicating.

The majority of the studies characterizing the urine microbiome in association with urological disorders focus on female subjects, as lower urinary tract symptoms are more common in adult females than males. Given anatomical differences in male and female urinary tracts, it is feasible to hypothesize that there would also be differences in the urinary microbiota. While not meeting statistical significance, it was interesting that there were differences identified in the bacterial biota of males and females regardless of infection status (Table 2 and Supplemental Figure 6). Much of the differences may reflect the uniqueness of the female genitourinary tract, where the vagina also has its own unique microbiota (Srinivasan et al., 2010) that potentially overlaps with the urinary tract. Unlike our prior studies in the human oral cavity (Abeles et al., 2014), there was no association between host-sex and urine viral community membership (Table 2 and Supplemental Figure 6). This study included only 20 subjects, which we used to screen for potential differences in virome contents between the sexes. It remains a possibility that a much larger cohort could identify differences urine viromes differences based on host sex. There appeared to be substantial inter-individual variability between the viromes of the subjects studied, however, we believe that longitudinal rather than cross-sectional studies of human viromes provide a better overview of variation observed between individuals (Abeles et al., 2014).

Despite specific HPV types being associated with low and high-health risks, just a fraction of the over 170 types have been directly implicated with disease, and many may not be detected using routine methods (Chaturvedi et al., 2011). Negative results may lead to undiagnosed illnesses such as cancerous lesions; yet, negative results have also been implicated with the existence of novel HPV types that remain to be characterized and associated with diverse disease phenotypes (Johansson et al., 2013). High-throughput sequencing has been shown to successfully detect several different HPV types in HPV-negative condylomas, suggesting that it may be a reasonable approach to detect HPV in HPV-negative specimens (Johansson et al., 2013). Our detection of HPV genotypes in the urine viromes of 19 subjects whom previously had not been diagnosed with HPV infections suggests that HPV colonization of the urine may be relatively common. That most viromes mapped across different HPVs, suggests the viruses in our subjects share common features with multiple known HPV genotypes. The data presented in this study indicate that viral metagenomics on urine could augment current HPV diagnostic approaches (Pathak et al., 2014).

### Conflict of interest statement

The authors declare that the research was conducted in the absence of any commercial or financial relationships that could be construed as a potential conflict of interest.
