# Supplementary material for: The human urine virome in association with urinary tract infections
Source: Front Microbiol. 2015 Jan 23;6:14. doi: 10.3389/fmicb.2015.00014 (PMC4304238; doi:10.3389/fmicb.2015.00014)
Supplement: Supplementary file 1 [file Table1.PDF]

Supplemental Table 1. Numbers of virome reads and contigs from all subjects

Subject	Number of reads	Average length	GC Content (%)	Number of contigs	Average length	GC Content (%)
URP1	662,120	217	40.7	3,200	1,225	43.0
URP2	632,903	216	40.6	3,215	1,303	40.8
URP3	514,004	197	38.0	1,581	1,236	41.0
URP4	766,694	203	40.2	2,062	1,180	41.0
URP6	564,278	213	39.4	1,957	1,104	41.5
URP7	785,652	209	37.4	2,112	1,139	40.5
URP9	768,502	202	39.7	1,715	1,214	40.0
URP10	624,144	209	38.9	1,390	1,251	39.0
URP12	691,906	220	39.2	1,761	1,148	40.1
URP14	724,661	214	40.9	1,598	1,163	41.6
URN1	534,412	213	41.6	3,909	1,180	44.0
URN2	858,681	212	41.0	3,181	1,223	42.9
URN6	765,829	205	42.9	2,067	1,214	41.7
URN9	658,048	192	39.4	1,704	1,190	40.2
URN10	650,536	201	41.2	1,810	1,232	42.0
URN11	1,003,850	206	40.6	3,096	1,257	43.5
URN12	1,116,705	198	39.2	3,109	1,199	41.0
URN13	729,718	200	40.6	2,689	1,164	42.0
URN15	631,819	198	40.0	939	1,389	40.5
URN16	857,887	207	40.4	2,610	1,177	42.0

Supplemental Table 2: Subjects with reads mapping to HPVs

Subject	HPV types
**Positive urine cultures**	
URP1	48, 49, 101, 112, 128, 135
URP2	None
URP3	53, 96, 109, 60, 6b, 132, 167
URP4	48, 5
URP6	50, 179, 129, 132
URP7	4, 167, 179, 131, 178, 109,
URP9	4, 112, 128
URP10	16, 49, 178
URP12	178, 60, 121, 4, 88, 144, 140, 129, 166, 112, 135, 126, 116, 167, 101, 136, 137, 50, 16, 103, 108, 109, 34, 63, 154, 134, 132, 32, 49
URP14	50, 7
**Negative urine cultures**	
URN1	4, 9, 34, 90, 49, 112
URN2	49, 96, 9, 5, 92, 178, 34, 26, 101, 7, 112, 116, 109, 60, 136,32, 129, 4, 121, 53, 48, 131, 167, 108, 6b, 63, 166
URN6	49, 9, 96, 92, 5, 178, 16, 34, 53, 7, 129, 26, 121, 60, 136, 4, 109, 140, 135, 112, 134, 131, 32, 137
URN9	6b, 179, 5, 60, 128
URN10	32, 34
URN11	49, 109, 128, 154
URN12	92, 136, 90, 48, 34, 166
URN13	90, 9, 4, 126, 132, 140
URN15	132, 26, 63, 135
URN16	128, 4, 5, 101
