# Supplementary material for: The human urine virome in association with urinary tract infections
Source: Front Microbiol. 2015 Jan 23;6:14. doi: 10.3389/fmicb.2015.00014 (PMC4304238; doi:10.3389/fmicb.2015.00014)
Supplement: Supplementary file 2 [file Presentation1.PDF]

Supplemental Figure 1

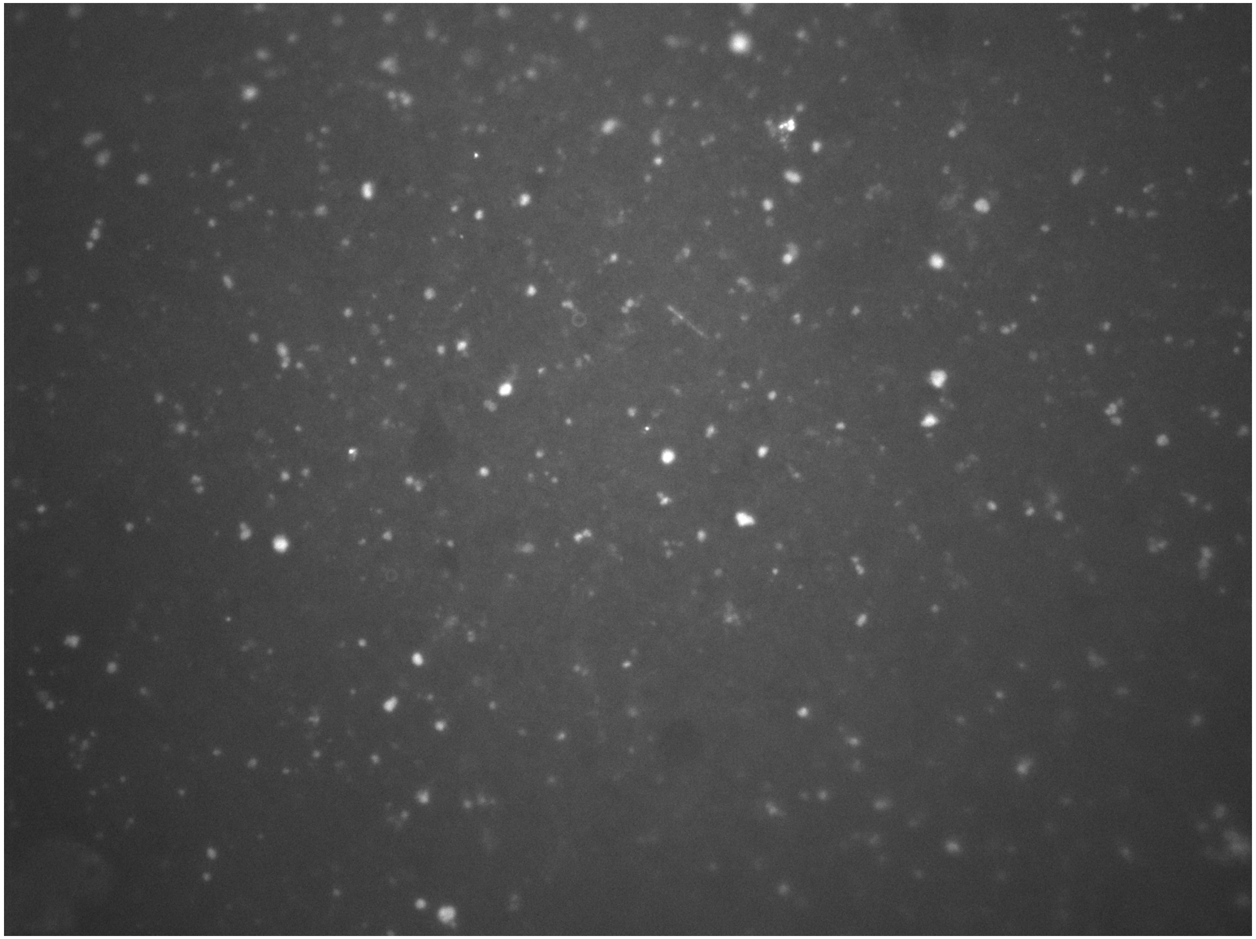

Supplemental Figure 1. Epifluorescence microscopy of virus-like particles (VLPs) from urine.

Supplemental Figure 2

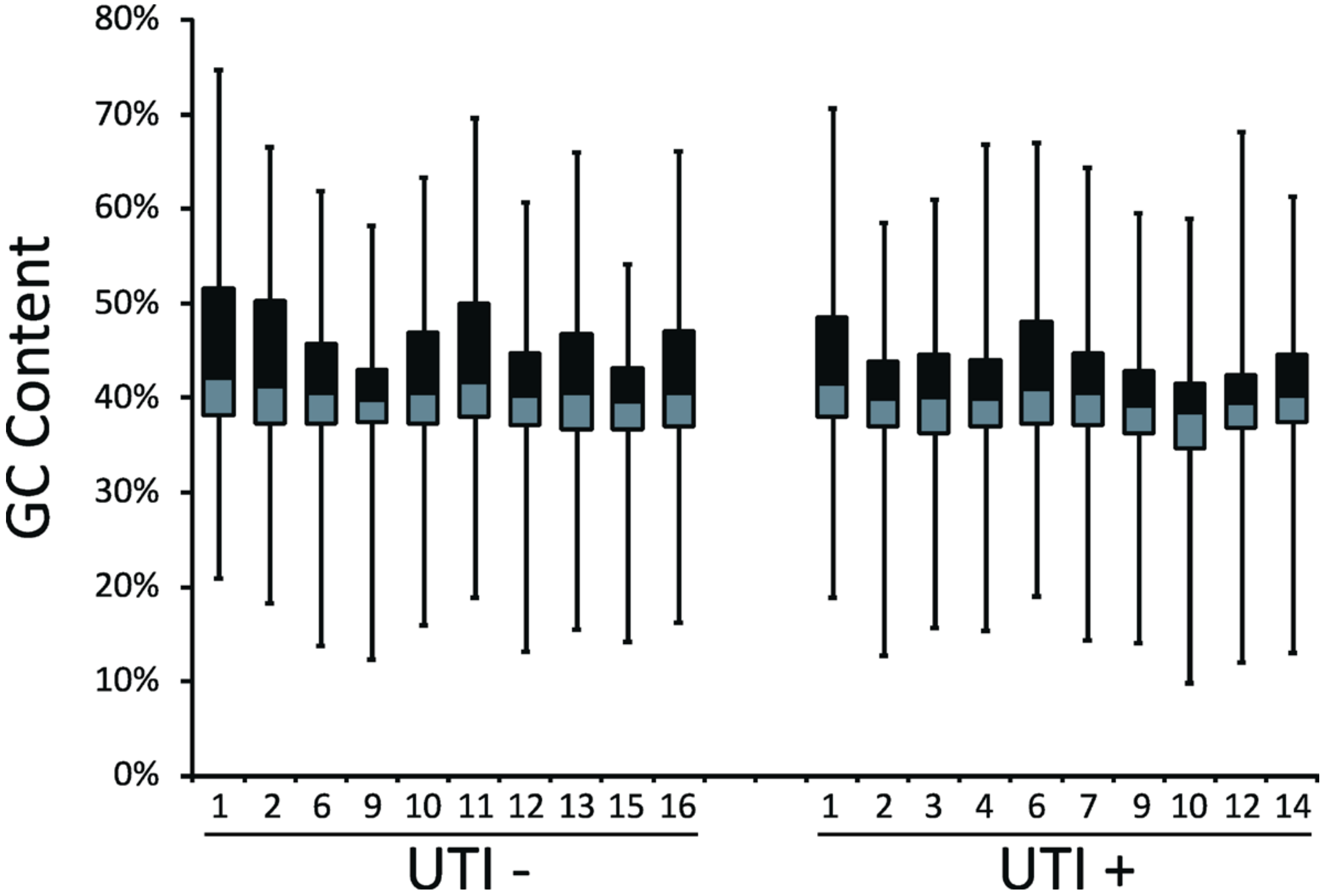

Supplemental Figure 2: Box and Whiskers plot of the median GC Contents of viral contigs in each subject. The gray boxes represent the third quartile, the black boxes represent the first quartile, and the second quartile (median) is represented at the intersection of the gray and black boxes. The error bars represent the minimum and maximum of all data points. GC content is demonstrated on the y-axis, and each subject is demonstrated on the x-axis.

Supplemental Figure 3

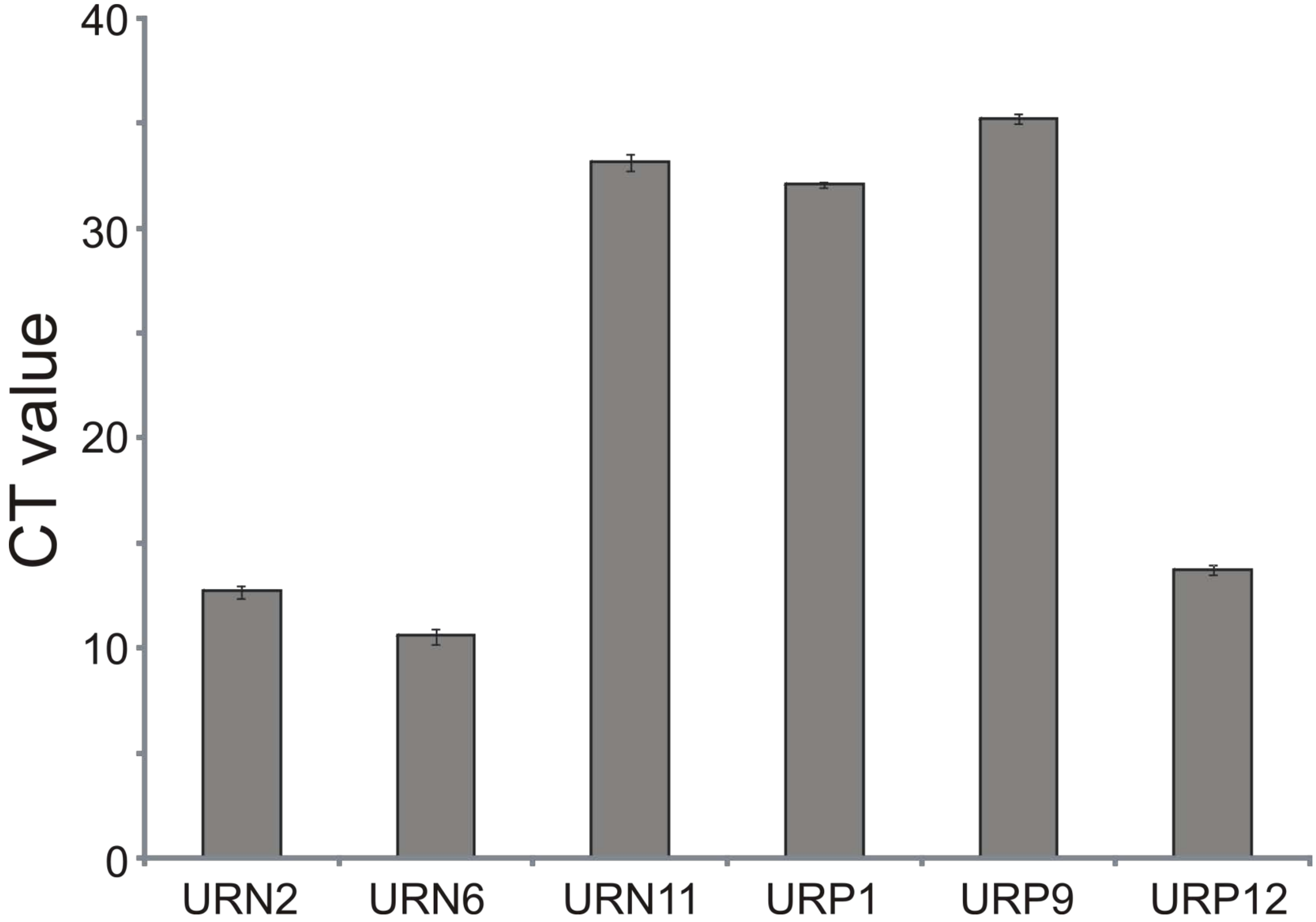

Supplemental Figure 3. Bar graphs of mean Ct values (±standard error) produced from 3-replicates of qPCR for HPV type 49.

Supplemental Figure 4

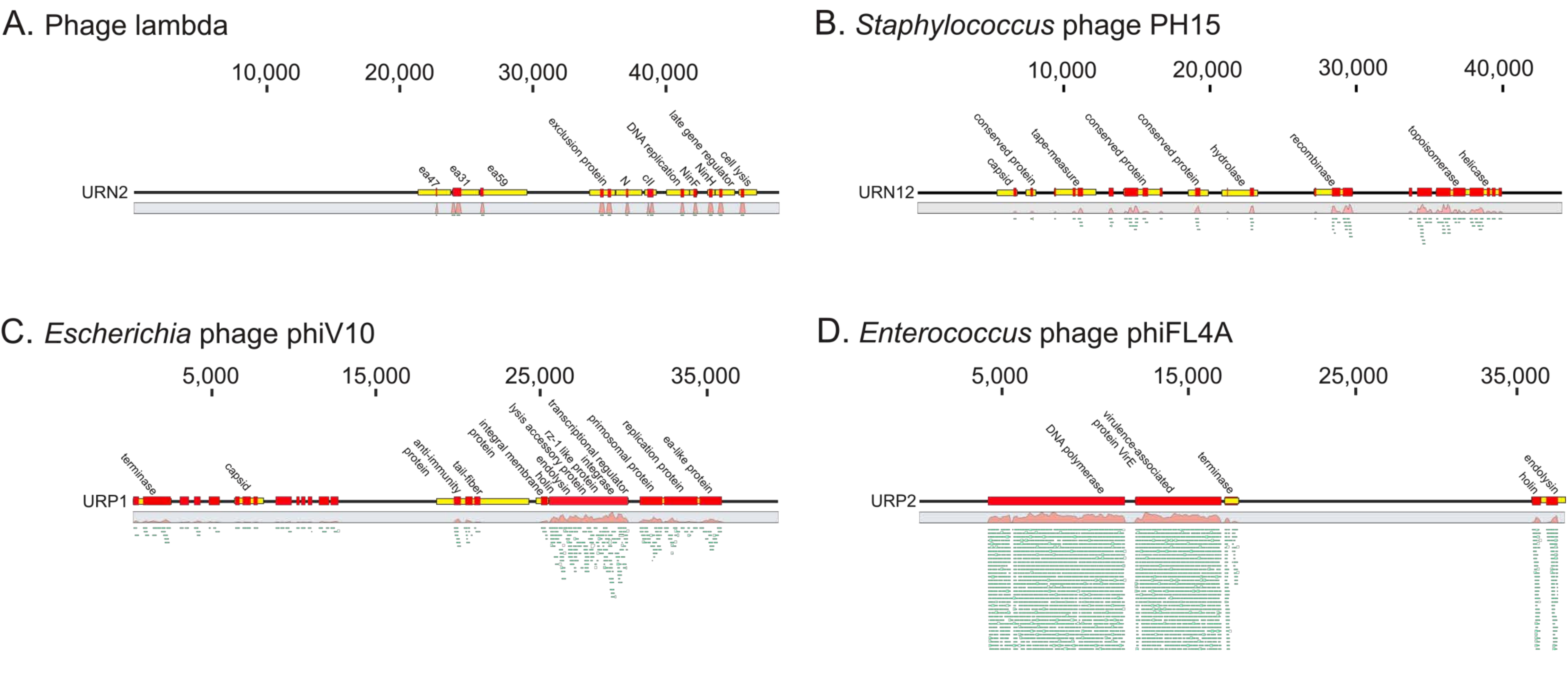

Supplemental Figure 4. Read mappings of select viromes to known bacteriophage. Panel A represents subject URN2 mapping to Lambda Phage, Panel B represents URN12 mapping to Staphylococcus phage PH15. Panel C represents URP1 mapping to E. coli phage phiV10, and Panel D represents URP2 mapping to Enterococcus phage phiFL4A. The relative coverage of each contig is represented, along with annotated open reading frames (ORFs) above each box. The portions of the contigs identified are represented by the colored boxes for each subject. The length of each contig is denoted at the top of each panel. The relative proportions of reads mapping to each virus are represented in green.

Supplemental Figure 5

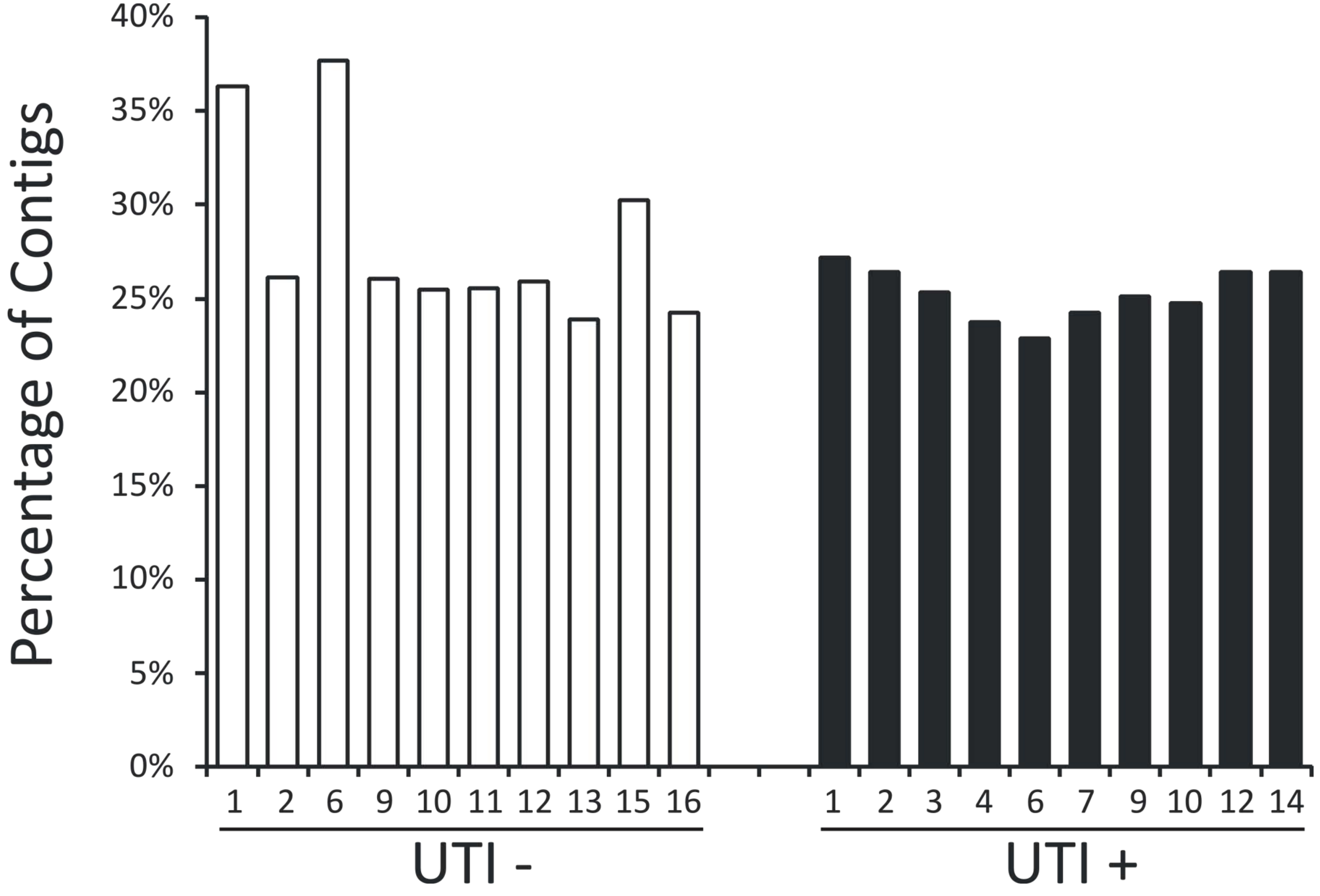

Supplemental Figure 5: Bar graph of the percentage of contigs with viral homologues in the NR database from all subjects.

Supplemental Figure 6

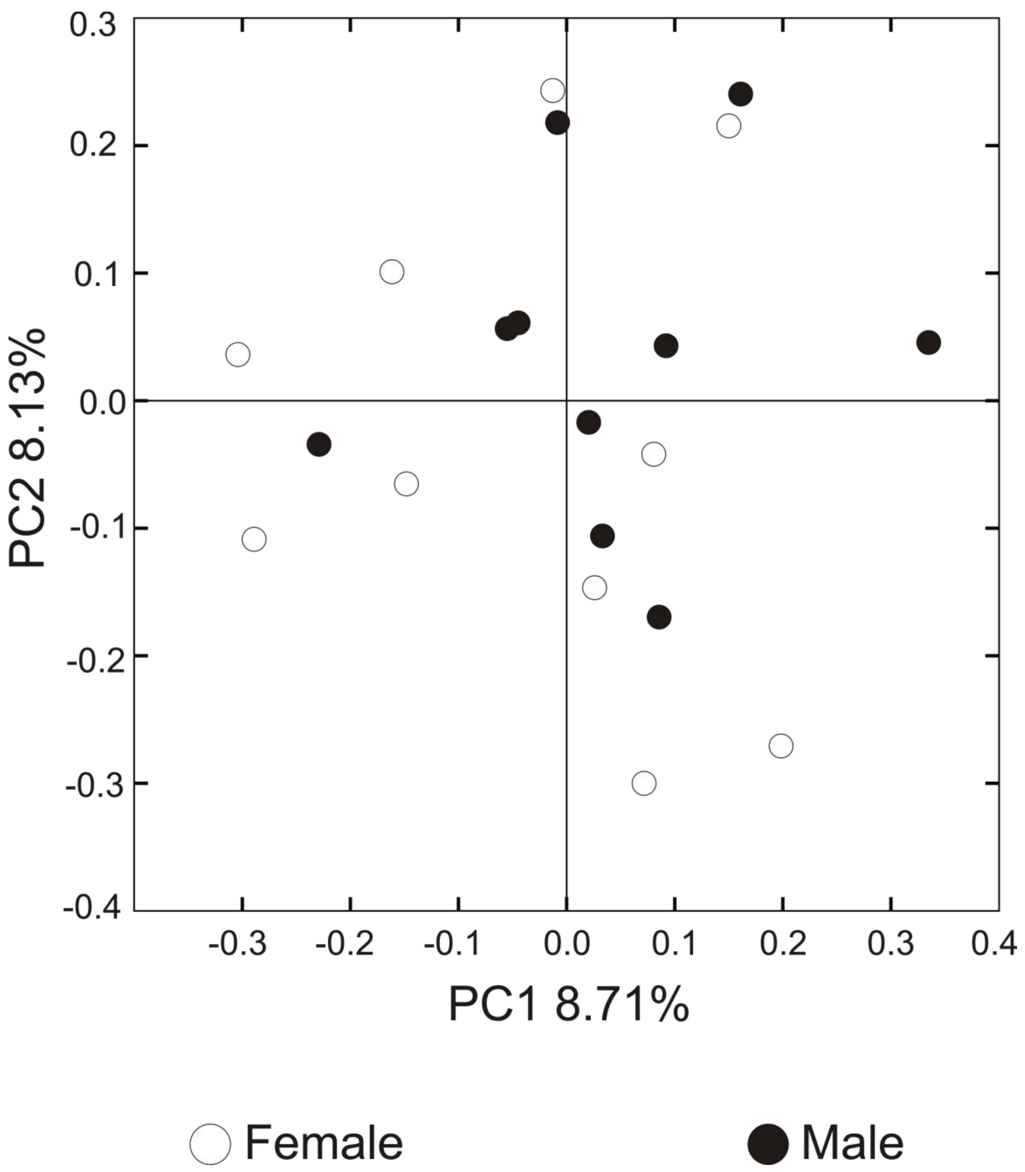

Supplemental Figure 6. Principal coordinates analysis of beta diversity of the urine viromes. White circles indicate female subjects and black circles represent male subjects.

Supplemental Figure 7

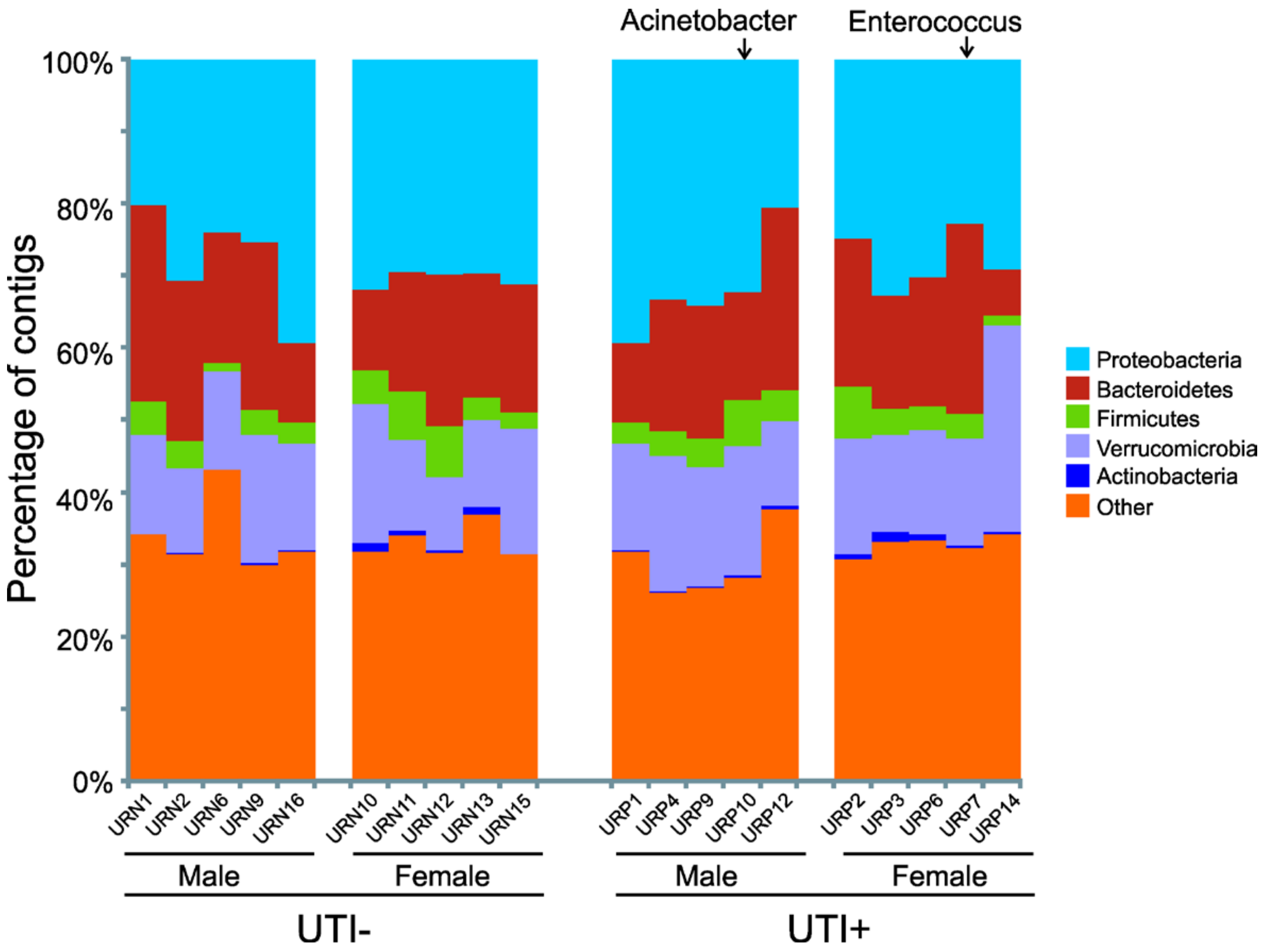

Supplemental Figure 7. Bar graphs of phylum-level BLASTX hits from the viromes of all subjects. The percentage of contigs with BLASTX hits homologous to different phyla is represented on the y-axis, and UTI+ and UTI- subjects are shown based on their sex on the x-axis.
